# Identification of Burkholderia cepacia Complex: Comparing Conventional, Automated, and Molecular Methods in a Tertiary Care Center

**DOI:** 10.7759/cureus.70847

**Published:** 2024-10-04

**Authors:** Priya Periaiah, Tessa Antony, Senita Samuel

**Affiliations:** 1 Department of Microbiology, Sri Ramachandra Institute of Higher Education and Research, Chennai, IND

**Keywords:** burkholderia cepacia complex, groel, indwelling devices, lysine decarboxylase, maldi-tof ms, minimum inhibitory concentration, polymyxin b

## Abstract

Introduction

*Burkholderia cepacia *complex(BCC) is one of the most common polymyxin-resistant Gram-negative bacilli isolated in the clinical microbiological laboratory. They are often underreported when conventional biochemicals are used for identification, due to their similarity to other non-fermenting bacilli. It is essential to identify BCC using simple biochemical tests with good reliability to ease the identification of BCC in resource-limited settings and initiate treatment.

Objectives

We aim to identify isolates belonging to BCC from other non-fermenters using simple conventional biochemical tests, automated methods, and polymerase chain reaction (PCR) and analyze antibiotic profiles and treatment outcomes in patients with BCC infection.

Materials and methods

All samples received at the clinical microbiology laboratory for bacterial culture from March 2023 to March 2024 were included in this study. Samples that grew non-lactose fermenting colonies on MacConkey agar, which were resistant to polymyxin B 300 (PB300) units, were further identified using conventional biochemicals, and lysine decarboxylase test, ornithine decarboxylase test, arginine dihydrolase test, ortho-nitrophenyl-β-galactoside (ONPG) test, urea hydrolysis, triple disc screening test (polymyxin B 300 units, amoxicillin-clavulanic acid 20/10 mcg (AMC20/10), and gentamicin 10 mcg (GEN10)), VITEK MS (bioMérieux, Marcy-l'Etoile, France), VITEK 2 Gram-Negative (GN) Identification (ID) Card (bioMérieux), and PCR targeting *groEL* sequence was performed for *Burkholderia* isolates. The antibiotic susceptibility pattern was analyzed using VITEK AST (bioMérieux). Basic patient details were collected from the medical records.

Results

Using conventional biochemicals and automated methods, 60 isolates were identified belonging to the genus *Burkholderia*, among which 46 (76.6%) belonged to *Burkholderia cepacia *complex. The concordance for genus-level identification of *Burkholderia *with conventional biochemicals, VITEK MS, VITEK 2, and *groEL *PCR was found to be 100%. Species-level identification using the listed conventional biochemicals was 28.2% as compared to VITEK MS.All the isolates were susceptible to ceftazidime (n=46, 100%). Risk factors included diabetes mellitus (n=19, 41.3%) and indwelling devices such as central venous catheters (CVCs), urinary catheters, and mechanical ventilation. Recovery was seen in 40 (86.9%) patients on treatment with recommended antibiotics.

Conclusion

Our study shows that the identification of the genus *Burkholderia *by conventional biochemicals was as efficient as automated and molecular methods. Species identification of BCC was better with automated systems. Infections with BCC commonly occurred in patients with diabetes mellitus and indwelling devices. Treatment with recommended antibiotics showed high recovery rates.

## Introduction

*Burkholderia* are aerobic, motile, Gram-negative bacteria. They are intracellular pathogens and can affect both phagocytic and non-phagocytic cells of the host [1]. The notable members of the genus are *Burkholderia cepacia *complex (BCC), *Burkholderia pseudomallei*,* *and* Burkholderia gladioli*. *Burkholderia cepacia* complex comprises nine genetically closely related species and hence are named genomovars. The species in genomovars I to IX are *B. cepacia*,* B. multivorans*,* B. cenocepacia*,* B. stabilis*,* B. vietnamiensis*,* B. dolosa*,* B. ambifara*,* B. anthinia*,* *and* B. pyrrocinia*,* *respectively [2]. The members of this genus are intrinsically resistant to the polymyxin group of drugs. BCC is often associated with infections in patients with cystic fibrosis, and in patients without cystic fibrosis, it can cause infections after invasive procedures such as urethral instrumentation and indwelling central venous catheters (CVCs) [3]. BCC is also known to contaminate pharmaceutical products and can cause hospital outbreaks. Transmission of BCC is strain-dependent, and the most common species associated with infections in non-cystic fibrosis patients is *B. cepacia* [4,5]. In recent times, BCC has gained attention due to its ability to affect non-cystic fibrosis patients and cause significant morbidity and mortality. However, there are no precise reports of the prevalence of BCC infections in non-cystic fibrosis patients in India, as they are often reported as *Pseudomonas* species [6]. It is essential to accurately diagnose BCC infections as they can cause bacteremia, urinary tract infections, septic arthritis, and peritonitis in immunocompromised patients [6]. Diagnosing *Burkholderia* infections in the laboratory is a challenging task as they are misidentified as *Pseudomonas* species due to their similarities such as oxidase positive test, non-lactose fermenting colonies on culture, and producing a non-fermenter pattern of biochemicals [6].

Currently, the genus *Burkholderia* is suspected when isolates show resistance to polymyxin B, and accurate species are identified by automated and molecular methods. However, the genus *Burkholderia* and, to a certain extent, its species can be identified by a simple battery of conventional biochemicals and antibiotic discs, which can be utilized in resource-limited settings for the identification of BCC. Hence, our study was put forward to identify isolates belonging to the genus *Burkholderia* from other non-fermenters, identify BCC using simple conventional biochemical tests, compare it with automated methods, and confirm it using PCR, and devise a simple algorithm to facilitate the identification of BCC in resource-limited settings.

## Materials and methods

Study design and setting

This prospective study was conducted over one year from March 2023 to 2024 in the Department of Microbiology, Sri Ramachandra Institute of Higher Education and Research, Chennai, and was approved on March 28, 2023, by the Institutional Ethics Committee (IEC number: CSP-MED/23/MAR/85/61).

Sample processing and microbial identification

Samples received during the study period for aerobic bacterial culture were taken up for this study. This included blood, urine, pus, tissue, endotracheal secretion, and pleural fluid. The samples were cultured on blood agar, chocolate agar, and MacConkey agar and were incubated at 37°C. Plates were routinely examined for growth for five days. The growth of non-lactose fermenting colonies on MacConkey agar was taken up for this study. Samples such as urine, endotracheal secretion, and bronchoalveolar lavage were processed as per significant colony counts [7-10].

Conventional tests to identify BCC species

All non-lactose fermenting Gram-negative bacilli that grew on MacConkey agar were processed using conventional biochemicals (indole, triple sugar iron (TSI) agar, Simmon's citrate agar, Christensen's urea agar, mannitol motility medium, and phenylalanine deaminase agar). Additionally, esculin hydrolysis, lysine decarboxylase, ornithine decarboxylase, arginine dihydrolase, motility, and oxidase tests were performed for all polymyxin B-resistant isolates. Biochemical tests were performed using standard methods [4]. According to previous literature, the polymyxin B disc diffusion method for initial screening purposes in diagnostic laboratories has been considered useful for speciation [11].

A triple disc screening test was performed using the Kirby-Bauer disc diffusion method using polymyxin B 300 (PB300), amoxicillin-clavulanate 20/10 mcg (AMC20/10), and gentamicin 10 mcg (GEN10) antibiotic discs. As there are no zone breakpoints for BCC for the abovementioned drugs, *Pseudomonas* and Enterobacterales were used as standards [12]. A zone of inhibition for amoxicillin-clavulanic acid 20/10 mcg of ≥18 mm was considered susceptible and ≤13 mm was considered resistant, and for gentamicin 10 mcg, ≥15 mm was considered susceptible and ≤12 mm was considered resistant. Any zone of inhibition was considered susceptible to polymyxin B. BCC shows resistance to AMC20/10, GEN10, and PB300, which helps in differentiating it from *Burkholderia pseudomallei *and* Burkholderia gladioli.*

Automated identification of BCC species

All the isolates were also subjected to identification by VITEK MS (bioMérieux, Marcy-l'Etoile, France), which uses matrix-assisted laser desorption ionization-time of flight (MALDI-TOF) technology for identification of clinical isolates and by VITEK 2 Gram-Negative (GN) Identification (ID) Card (bioMérieux), which uses advanced colorimetry based on biochemicals, an identification technology that enables identification of clinical isolates. It identifies the species of *Burkholderia cepacia* complex (*B. cepacia*,* B. multivorans*,* B. vietnamiensis*, and* B. stablis*) as *Burkholderia cepacia* group as the database of VITEK 2 GN ID CARD system has them as a grouped taxa.

Molecular confirmation of the genus *Burkholderia*

Genomic DNA was extracted using the LUPEX BIO Bacterial DNA Isolation Kit (Lupex Biotech, Chennai, India). DNA extraction was done based on the manufacturer's instructions. A polymerase chain reaction was performed using primers targeting *groEL*. The primers were selected based on previously published literature [13]. The *groEL* gene encodes for an immunogenic protein of the genus *Burkholderia* that assists in proper protein folding and is highly conserved among *Burkholderia* species (Table 1).

**Table 1:** Genus-specific primers for Burkholderia targeting the groEL gene Forward and reverse nucleotide sequence of the *groEL* primer

Primer name	Nucleotide sequence(5’-3’)	Target gene	Amplicon size
Gro1-Forward	CTGGAAGACATCGCGATC	groEL (*Burkholderia* genus-specific)	139 bp
Gro2-Reverse	CGTCGATGATCGTCGTGTT		

Antibiotic susceptibility testing (AST)

Antibiotic susceptibility testing (AST) was performed using VITEK, which analyzes minimum inhibitory concentration and detects phenotypes. VITEK 2 AST-N406 card was used in our study. The minimum inhibitory concentrations of ceftazidime, meropenem, levofloxacin, trimethoprim-sulfamethoxazole (TMP-SMX), and minocycline were analyzed. Quality control was performed using *Pseudomonas aeruginosa* ATCC 27853 [12].

Basic demographic details and history were taken for samples collected during the routine course of patient care. Additional data related to hematological parameters and indwelling devices were collected during the hospital course from the hospital data system and medical records.

Statistical analysis

The data generated was entered into Microsoft Excel (Microsoft Corp., Redmond, WA) and assessed for statistical significance using IBM SPSS software version 17 (SPSS Inc., Chicago, IL). However, as the cross-sectional pilot study involved a lesser number of isolates, no statistically significant data could be generated.

## Results

Among 3,257 non-fermenting Gram-negative bacilli isolated on MacConkey agar, 105 (3.2%) isolates were polymyxin B-resistant. Polymyxin B-resistant non-fermenting Gram-negative bacilli growing on MacConkey agar included *Burkholderia*,* Myroides*,* Ralstonia*,* *and *Chryseobacterium* species. Using additional tests such as oxidase, motility, and other amino acid-based biochemicals, organisms belonging to BCC were identified. Among the 105 isolates, 72 (68.5%) were motile and 33 (45.8%) were non-motile. All *Burkholderia* species are motile except *Burkholderia mallei* (primary animal pathogen). Hence, all non-motile organisms were excluded from further identification. Among the 72 motile isolates, the oxidase test was positive for 69 (95.8%) isolates and negative for three (4.1%) isolates. Irrespective of the oxidase test, the lysine decarboxylase test, ornithine decarboxylase test, and arginine dihydrolase test were performed on all 72 motile isolates, as the genus *Burkholderia *contains both oxidase positive and negative species (Table 2). 

**Table 2:** Identification of the genus Burkholderia and BCC from other motile polymyxin B-resistant isolates + represents positive, and - represents negative. BCC: *Burkholderia cepacia* complex

Oxidase	Lysine decarboxylase	Arginine dihydrolase	Number of isolates	Percentage of isolates	Interpretation
Positive	+	-	46	63.8%	BCC
Positive	-	+	11	15.2%	*Burkholderia pseudomallei* (excluded from the study)
Positive	-	-	12	16.6%	*Ralstonia* species (excluded from the study)
Negative	-	-	3	4.16%	*Burkholderia gladioli* (excluded from the study)

A total of 60 isolates belonging to the genus *Burkholderia* were identified, among which only 46 (76.6%) isolates identified as BCC were further speciated using urea hydrolysis, esculin hydrolysis, and ortho-nitrophenyl-β-galactoside (ONPG) tests.

**Figure 1:**
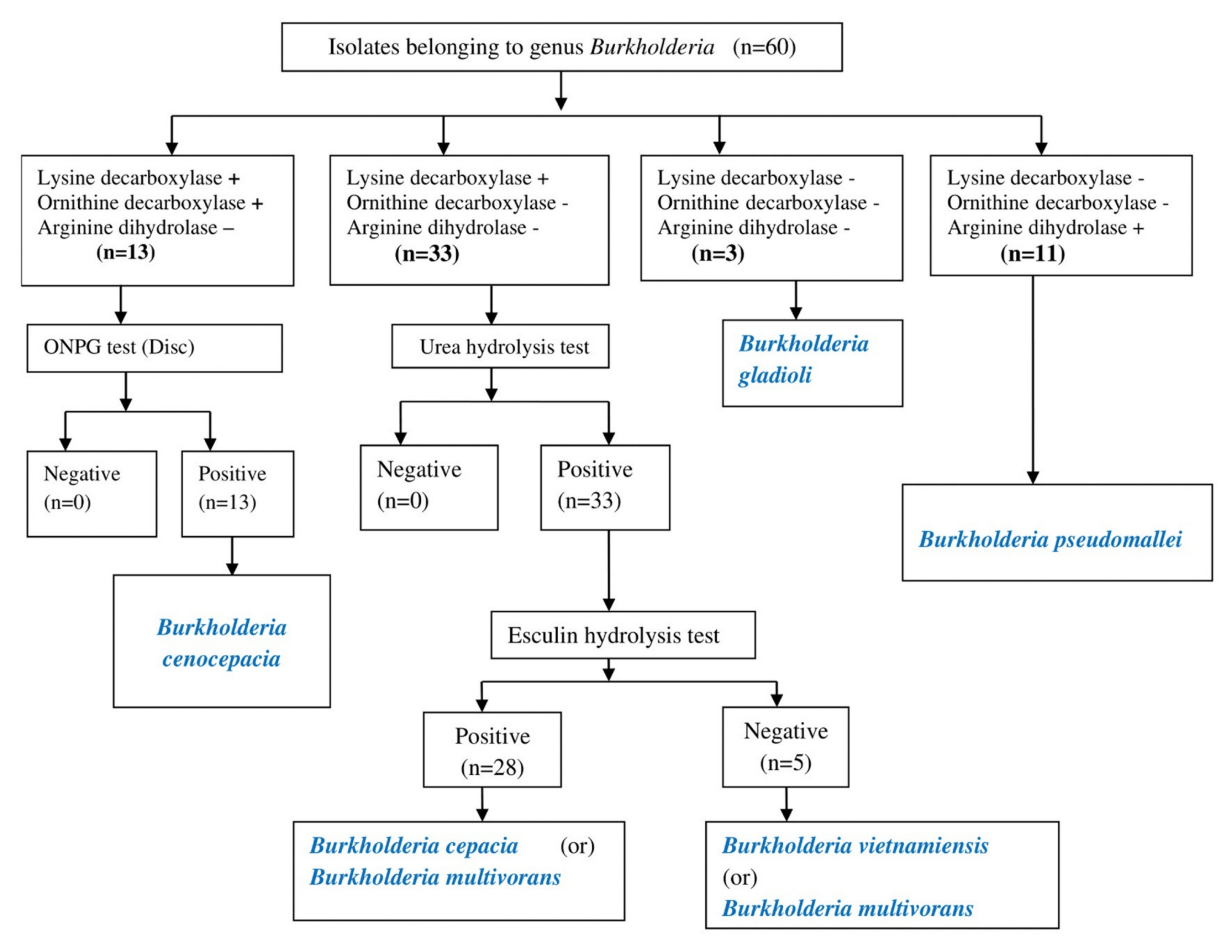
Identification of Burkholderia species including intra-species identification of BCC The algorithm was designed based on Koneman's Color Atlas and Textbook of Diagnostic Microbiology [4]. + represents positive, and - represents negative. n represents the number of isolates. BCC: Burkholderia cepacia complex, ONPG: ortho-nitrophenyl-β-galactoside

Among the 46 isolates of BCC, *Burkholderia cenocepacia* (n=13, 28.2%) were differentiated from other BCC species as they were lysine decarboxylase-positive, ornithine decarboxylase-positive, and ONPG test-positive. The remaining 33 (71.7%) isolates of BCC could not be differentiated with the use of a selected panel of conventional biochemicals (Figure 1).

**Figure 2:**
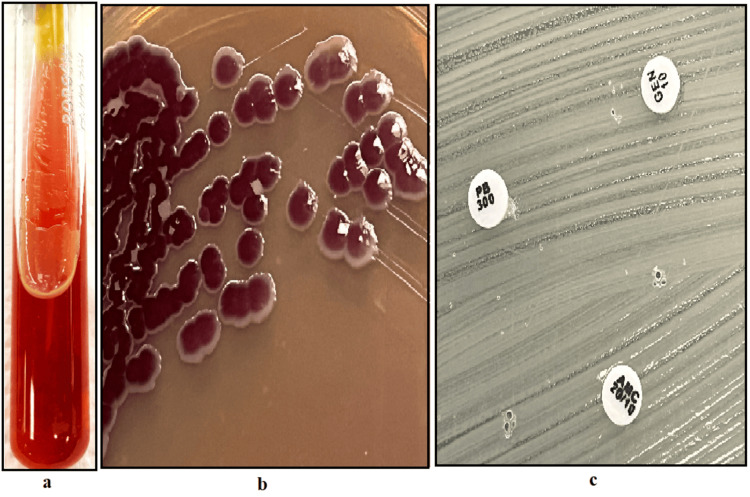
Phenotypic characteristics of Burkholderia cepacia complex Figure 2a shows a bright yellow pigment produced by *Burkholderia cepacia* complex on triple sugar iron agar. Figure 2b shows purple-colored colonies of *Burkholderia cepacia* on MacConkey agar. Figure 2c shows triple disc resistance exhibited by *Burkholderia cepacia* complex: resistance to polymyxin B, amoxicillin-clavulanic acid, and gentamicin discs.

Some of the isolates of* B. cepacia* also produced a bright yellow pigment on triple sugar iron agar, which helped in the identification of organisms belonging to other genera (Figure 2a). Two (4.3%) isolates belonging to BCC produced distinctive purple-colored colonies on MacConkey agar, which was later identified as *Burkholderia cepacia* by VITEK MS (Figure 2b). All the BCC isolates also showed resistance to triple disc testing by polymyxin B 300, AMC20/10, and GEN10; this was useful in the identification of BCC from *B. gladioli* and *B. pseudomallei *(Figure 2c).

All 46 isolates of BCC identified by conventional methods were also identified by automated methods using VITEK MS and VITEK 2 Gram-Negative Identification Card. Among the 46 isolates, genus-level identification by VITEK MS for *Burkholderia* was found to be 100% compared to PCR. VITEK MS identified 41 (89.1%) isolates of *Burkholderia cepacia *complex with a 99% confidence value, 22 (47.8%) of *B. cepacia*, 13 (28.2%) of *B. cenocepacia*, three (6.5%) of *B. multivorans*, and three (6.5%) of *B. vietnamiensis*. Identification with lesser confidence values was observed in five (10.8%) isolates; this included four isolates identified as *B. cepacia* or *B. multivorans *with 50% confidence and one isolate identified as *B. cepacia* or *B. multivorans* or *B. vietnamiensis* with 33% confidence (Table 3). Using VITEK 2 GN ID Card, in the 46 isolates of *Burkholderia cepacia *complex, genus-level identification was found to be 100%, and grouped taxa identification of BCC was found to be 100% (n=46), as all the isolates belonging to BCC were reported as *Burkholderia cepacia* group.

**Table 3:** Identification of Burkholderia cepacia complex species by VITEK MS with confidence values Confidence values given by VITEK MS are mentioned in percentages (%). MALDI-TOF: matrix-assisted laser desorption ionization-time of flight

MALDI-TOF identification	Confidence values (%)	Number of isolates	Percentage of isolates
Burkholderia cepacia	99.9%	22	47.8%
*Burkholderia cepacia* (or) *Burkholderia multivorans*	50%	4	8.6%
*Burkholderia cepacia* (or) *Burkholderia vietnamiensis* (or) *Burkholderia multivorans*	33%	1	2.17%
Burkholderia cenocepacia	99.9%	13	28.2%
Burkholderia multivorans	99.9%	3	6.5%
Burkholderia vietnamiensis	99.9%	3	6.5%

**Figure 3:**
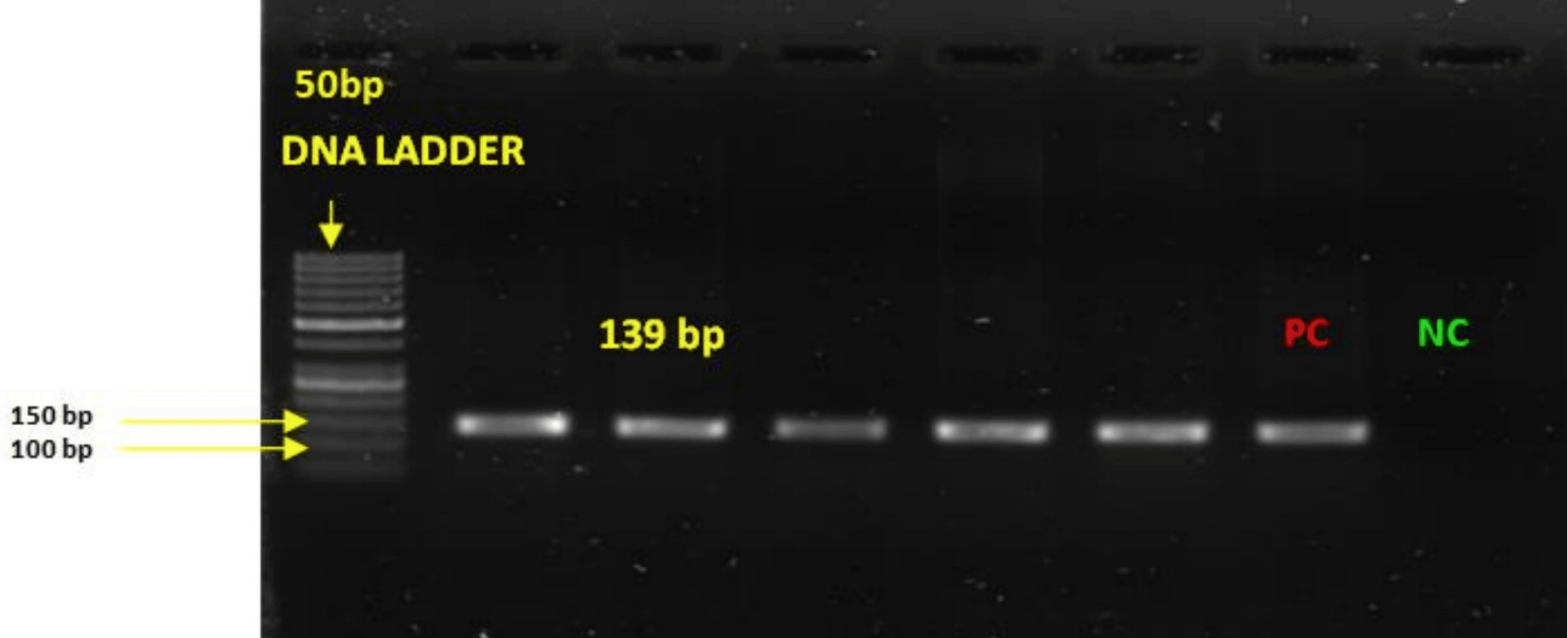
Gel image showing amplification for the genus Burkholderia using genus-specific primer targeting groEL (139 base pairs) PC represents positive control (*Burkholderia cepacia* isolate sequenced using 16s rRNA with GenBank accession number PP813440 was used as the positive control). NC represents negative control (nuclease-free water was used as the negative control). bp: base pairs

Molecular confirmation of the genus *Burkholderia* was done by PCR using primers targeting *groEL*. The expected amplicon size of 139 base pairs (bp) was seen in all 46 (100%) isolates (Figure 3).

**Figure 4:**
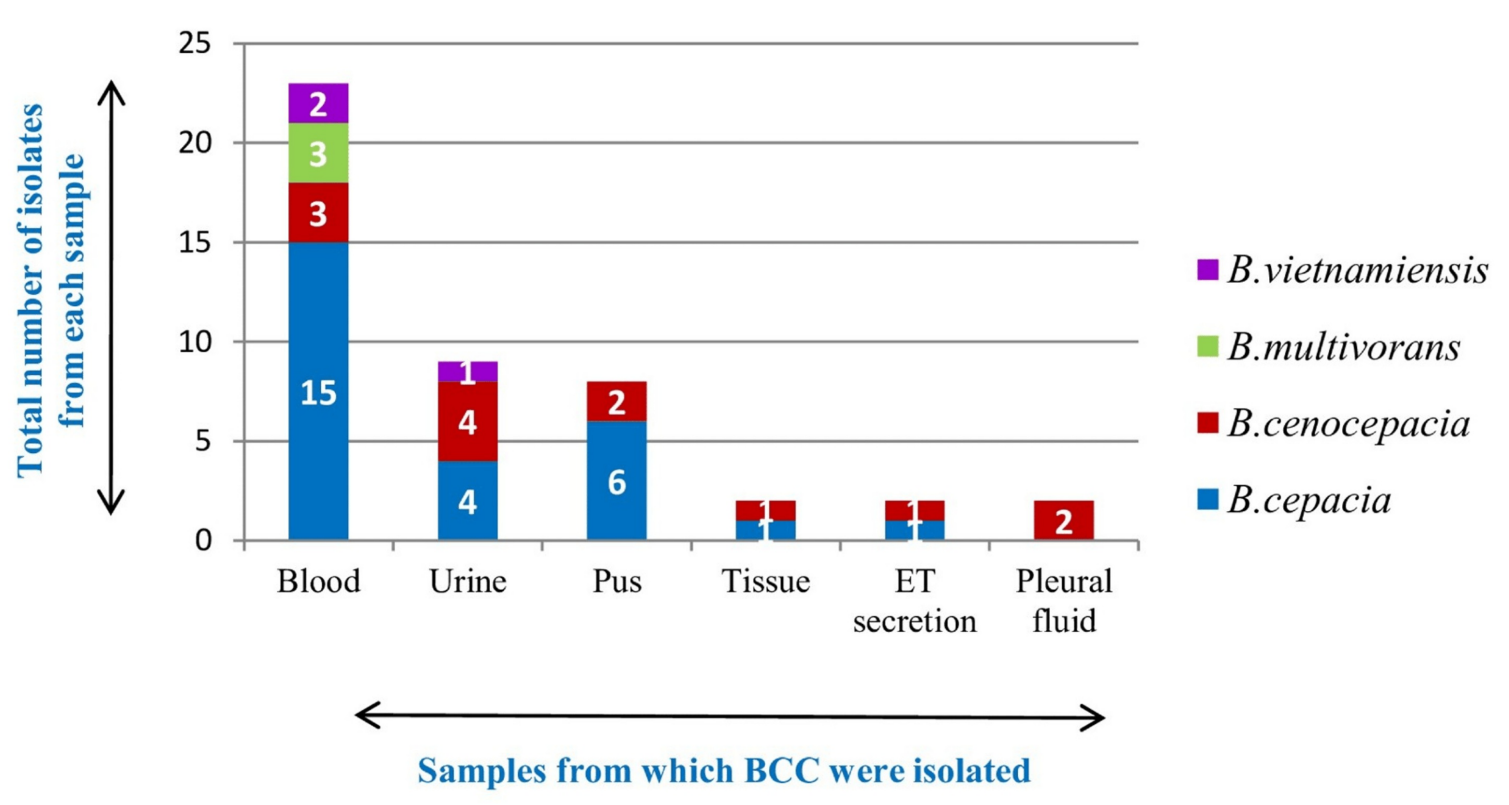
Sample and species distribution of Burkholderia cepacia complex BCC: *Burkholderia cepacia *complex, ET: endotracheal

BCC was isolated from blood in 23 (50%) patients, followed by pus and tissue samples in 10 (21.7%) patients, urine in nine (19.5%) patients, and respiratory samples in four (8.6%) patients (Figure 4). *Burkholderia cepacia* was the most common species isolated among the BCC (n=27, 58.69%). Among 46 patients with BCC infections, 31 (67.3%) patients were males and 15 (32.6%) were females. Patients above 18 years were 69.5% (n=32) and those below 18 years were 30.4% (n=14), out of which 15.2% (n=7) were infants.

**Figure 5:**
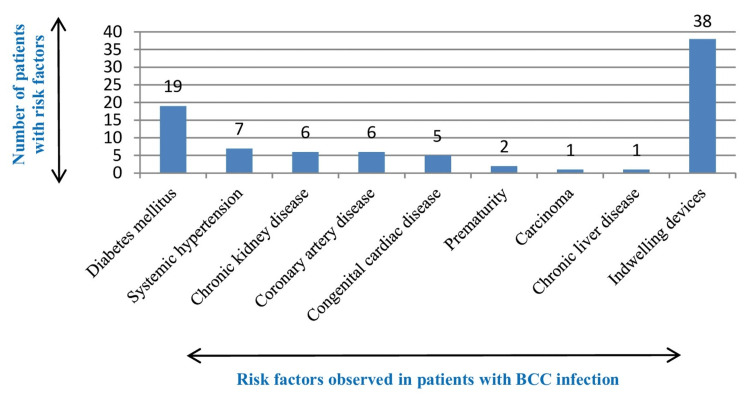
Risk factors in patients with Burkholderia cepacia complex infection BCC: *Burkholderia cepacia* complex

Among 46 patients with BCC infection, 30 (65.2%) patients were from the intensive care unit (ICU) and 16 (34.7%) were non-ICU patients. Overall, the risk factors observed in this study included diabetes mellitus, systemic hypertension, chronic kidney disease, chronic liver disease, coronary artery disease, prematurity, congenital cardiac disease, carcinoma, and indwelling devices (Figure 5). 

Among the patients in the ICU, multiple risk factors predominated (n=13), and in non-ICU patients, single risk factors were more commonly observed (n=10). A total of 14 (30.4%) patients, 13 ICU patients and one non-ICU patient, had more than one risk factor (Table 4).

**Table 4:** Multiple risk factors observed in 14 patients Among patients with multiple risk factors, 13 of them were ICU patients, and one patient with systemic hypertension and chronic kidney disease was a non-ICU patient. ICU: intensive care unit

Multiple risk factors	Number of patients
Diabetes mellitus with systemic hypertension	2
Diabetes mellitus with coronary artery disease	3
Diabetes mellitus with chronic kidney disease	2
Diabetes mellitus with systemic hypertension and coronary artery disease	1
Diabetes mellitus with systemic hypertension and chronic kidney disease	1
Systemic hypertension with chronic kidney disease	2
Systemic hypertension with coronary artery disease	1
Prematurity with congenital cardiac disease	2

Pertaining to indwelling devices, BCC bacteremia occurred in 50% (n=23) (seven (30.4%) patients with central venous catheters and 16 (69.5%) patients with peripheral venous lines). In patients with bacteremia, indwelling devices were present in 91.3% (n=21) and absent in 8.6% (n=2) of the patients. This included central venous catheters in 30.4% (n=7), urinary catheters in 56.5% (n=13), and mechanical ventilation in 78.2% (n=18). In the remaining 50% (n=23) of patients without bacteremia, BCC was isolated from urine in 26% (n=6) with urinary catheters who had undergone urethral instrumentation procedure, from pleural fluid in 8.6% (n=2) of patients with underlying lung condition with intercostal drain, and from endotracheal secretion in 8.6% (n=2) of patients with pneumonia. Additionally, BCC was also isolated from patients without site-specific indwelling devices (urine, 13.04% (n=3); skin and soft tissue, 43.4% (n=10)).

Antibiotic susceptibility testing in BCC isolates showed that all the isolates were susceptible to ceftazidime (n=46, 100%), followed by trimethoprim-sulfamethoxazole (n=42, 91.3%), meropenem (n=40, 86.9%), levofloxacin (n=37, 80.4%), and minocycline (n=36, 78.2%). Based on the AST profile, 34.7% (n=16) of the patients were treated with fluoroquinolones, 19.5% (n=9) with ceftazidime, 19.5% (n=9) with meropenem, 6.5% (n=3) with trimethoprim-sulfamethoxazole, and 2.17% (n=1) with minocycline. A total of eight (17.3%) patients were on combination therapy with fluoroquinolone and trimethoprim-sulfamethoxazole (n=2, 4.3%), ceftazidime and levofloxacin (n=2, 4.3%), meropenem and levofloxacin (n=3, 6.5%), and meropenem and trimethoprim-sulfamethoxazole (n=1, 2.17%). The duration of treatment with fluoroquinolones, ceftazidime, trimethoprim-sulfamethoxazole, and minocycline ranged between five and seven days and meropenem between seven and 21 days.

A total of 86.9% of the patients recovered (n=40), indicated by repeat sterile cultures, while 13.04% (n=6) of the patients with underlying comorbidities succumbed to the infection. In six (13.04%) patients who succumbed, we observed *Burkholderia cepacia* bacteremia; it was complicated by conditions such as severe pneumonia, congenital cardiac disease, coronary artery disease, chronic liver disease, hypovolemic shock, and septic shock.

## Discussion

*Burkholderia* are non-fermenting Gram-negative bacilli intrinsically resistant to polymyxin B. They are now being recognized as emerging pathogens. BCC is the fourth most commonly occurring non-fermenting Gram-negative bacilli, secondary to the ESKAPE (*Enterococcus faecium*, *Staphylococcus aureus*, *Klebsiella pneumoniae*, *Acinetobacter baumannii*, *Pseudomonas aeruginosa*, and *Enterobacter* spp.) group of organisms [14]. In India, 8.8% of hospital-acquired infection outbreaks occur due to BCC [15]. The most frequently isolated species in non-cystic fibrosis patients is *Burkholderia cepacia *[3,16]. Laboratory diagnosis of BCC infections is based on culture and biochemical identification. Routine biochemical tests can be used to identify these organisms to genus and species level as BCC, *Burkholderia gladioli*,* *and *Burkholderia pseudomallei*. However, intra-species identification among the BCC is a tedious task because *Burkholderia cepacia* complex is ever expanding with new pathogens getting included often [17]. The latest modalities, such as VITEK MS and VITEK Gram-Negative Identification Card, help in the rapid identification of the organism. However, identification by automated systems can be expensive and may not be available in all settings [18]. Therefore, it is essential to develop a simple algorithm for the identification of these bacteria using conventional biochemicals with good reliability so it can be used in all resource-limited settings.

Based on polymyxin B resistance and conventional tests such as oxidase, motility, lysine decarboxylase, ornithine decarboxylase, arginine dihydrolase, and triple antibiotic discs (PB300, AMC20/10, and GEN10), 60 isolates belonging to the genus *Burkholderia* were identified from other non-fermenters (100%). This aligns with the study done by Gautam et al., where the identification rate using conventional biochemicals for non-fermenters was 99% [6]. The abovementioned tests also helped in the differentiation of *Burkholderia cepacia *complex (n=46) from *Burkholderia gladioli* (n=3) and *Burkholderia pseudomallei* (n=11). This was also consistent with the study done by Henry et al., where they were able to differentiate all isolates of *Burkholderia cepacia* complex from *B. gladioli* using routine biochemical tests [17].

The incidence of BCC was 1.2% among the non-fermenters in our study. Among the 46 isolates belonging to *Burkholderia cepacia* complex, we were only able to speciate *Burkholderia cenocepacia* (13 isolates, 28.2%) with routine biochemicals used in the microbiological laboratory. Henry et al., in their study, could not differentiate between the species of BCC even with the use of an extensive panel of biochemicals, whereas in our study, we were able to speciate *Burkholderia cenocepacia *[17]. Some of the isolates of *B. cepacia *produced bright yellow-colored pigment on triple sugar iron (TSI) agar, and two isolates (4.3%) produced purple-colored pigment on MacConkey agar, which helped in the identification of *B. cepacia* from other species of the same genus. Hence, our study emphasizes the need to suspect BCC when a purple pigment colony or bright yellow pigment on TSI agar is produced. It is important to keep in mind these characteristic findings, which can be an added factor to conventional biochemicals in the identification of BCC. BCC-producing purple pigment was reported in studies from North India by Rastogi et al. [19] and De et al. [20]. To the best of our knowledge, our study is one of the first to report this distinct purple-colored pigment produced by *B. cepacia* from South India. Further studies are required to identify the factors causing such pigments in BCC and its association with virulence.

All the isolates were subjected to automated testing by VITEK MS and VITEK 2 GN ID Card. The genus-level identification of *Burkholderia *by both methods was 100%. Species identification of *Burkholderia cepacia* complex with 99% confidence was observed to be 89.1% (n=41). This was in accordance with a study done by Fehlberg et al., who observed 76.9% species-level identification [21]. A total of five (10.8%) isolates of BCC were identified with lower confidence of 50% and 33%. This finding was similar to a study done by Wong et al., in which 10 isolates of BCC were observed with lesser confidence [18]. VITEK 2 identifies BCC species as a grouped taxa; hence, they were identified as *Burkholderia cepacia *group as per the database. In our study, VITEK MS was found to be highly efficient in identifying most of the BCC species. Molecular confirmation of the genus *Burkholderia* for all the BCC isolates was found to be concordant with VITEK 2. Our study shows that the identification of the genus *Burkholderia* was reliable by using conventional biochemicals, VITEK MS, VITEK 2, and PCR. Species identification was more accurate with VITEK MS.

All 46 patients in our study were non-cystic fibrosis patients. Cystic fibrosis is considered a rare entity in India and is often underdiagnosed. Among 46 patients, 14 (30.4%) had more than one risk factor. Diabetes mellitus (41.3%) was the most common risk factor observed in adults. Other risk factors such as hypertension, chronic kidney disease, and chronic liver disease were also identified, but there was no adequate correlation to infection acquisition. In neonates and infants with congenital cardiac disease (n=5, 10.8%), the use of central venous catheters was one of the most common risk factors observed. This finding was similar to a study done by Cetin et al., who described that in infants with congenital defects, prolonged use of central venous lines is a risk factor for BCC acquisition [22]. BCC is an opportunistic infection, and in an immunocompromised host such as those with diabetes mellitus, chronic kidney disease, and congenital cardiac disease, it can cause infections due to hyperglycemia promoting bacterial growth, impairing innate immune cell function, and the use of central venous catheters enables BCC to colonize and invade. With BCC being intrinsically resistant to various groups of drugs, limited therapeutic options are available, leading to morbidity and mortality.

BCC bacteremia occurred in 78.2% of those who were on mechanical ventilation, 56.5% had urinary catheters, and 30.4% of the patients had central venous catheters. Other than blood, BCC was also isolated from respiratory samples such as endotracheal secretion (n=2, 8.6%) and pleural fluid (n=2, 8.6%) and in six (26%) patients from urine. BCC bacteremia was associated with patients having prolonged indwelling devices such as CVC, mechanical ventilation, and urinary catheters. Respiratory infections with BCC were shown to be more in patients with prolonged mechanical ventilation, the presence of an intercostal drain, and prolonged stay in the ICU. BCC UTIs were shown to occur more in patients who had undergone urethral procedures and had urinary catheters. Given that these are common risk factors seen in patients in the ICU settings, it is essential to assess for early removal of such devices to mitigate the risk of biofilm formation. In a study done by Abdallah et al., they observed that indwelling devices such as central venous catheters and mechanical ventilation were a few of the major risk factors contributing to BCC bacteremia [3]. Pegues et al. [23] and Hamill et al. [24] reported that BCC was isolated from respiratory samples in patients who were on mechanical ventilation for two days or more or were intubated more than once and had received nebulization therapy. In a study done by Du et al. in patients with BCC UTI, indwelling catheters and invasive urethral procedures were significant risk factors [25].

The antibiotic susceptibility trend in our study showed that all the BCC isolates were susceptible to ceftazidime (n=46, 100%), followed by trimethoprim-sulfamethoxazole (TMP-SMX) (n=42, 91.3%), meropenem (n=40, 86.9%), levofloxacin (n=37, 80.4%), and minocycline (n=36, 78.2%). We observed that most of the patients were prescribed fluoroquinolones, followed by ceftazidime. This finding was similar to a 17-year nationwide study on BCC done by El Chakhtoura et al., who observed that fluoroquinolones were the most commonly prescribed antibiotic in treating BCC infections [26]. We observed that combination therapy with dual drugs was given for 17.3% of patients (n=8) after laboratory reporting of BCC infection. In our study, the most commonly used agent in dual combination therapy was meropenem with TMP-SMX or fluoroquinolone. Similarly, Avgeri et al. reported that meropenem was the most commonly used agent for combination therapy and was found to be effective [27]. While treatment with a single drug regimen had a 84.2% (n=40) recovery rate, all patients treated with combination therapy with at least two drugs showed 100% (n=8) recovery. In total, 86.9% (n=40) of the patients recovered when treated with the recommended antibiotics, irrespective of the regimen. Based on antibiotic susceptibility testing, it is essential to treat BCC infections with recommended antibiotics, as they are intrinsically resistant to penicillins, aminoglycosides, and polymyxins.

Limitations

A multicenter study involving more isolates and an extended duration of study could have resulted in statistically significant results. A long-term study would have helped in better analysis of specific risk factors associated with BCC infection acquisition, antibiotic susceptibility trends, and patient outcomes. While we successfully identified *Burkholderia* at the genus level and distinguished between *Burkholderia cepacia *complex, *B. pseudomallei*, and *B. gladioli*, we were unable to identify all the species of BCC with the use of a routine panel of biochemicals.

## Conclusions

*Burkholderia cepacia *complex are emerging pathogens in India. Our study emphasizes that diabetes mellitus and indwelling devices such as central venous catheters, urinary catheters, and mechanical ventilation should still be considered important risk factors in BCC infection acquisition. Our study shows that the use of conventional biochemical tests such as oxidase, motility, lysine decarboxylase, ornithine decarboxylase, and triple disc screening are as efficient as automated and molecular methods in identifying BCC from other non-fermenters. The antibiotic susceptibility trend in our study shows that the resistance rates for recommended antibiotics were low. The commonly used antibiotics to treat BCC (ceftazidime, meropenem, fluoroquinolones, and TMP-SMX) either as monotherapy or in combination showed good recovery rates. Accurate identification and reporting of BCC infections are crucial, as they can lead to significant morbidity and mortality. Early identification by automated methods and performing PCR directly on samples in combination with strict infection control protocol can reduce the spread of BCC infection and thereby morbidity and mortality. In resource-limited settings, automated identification may not always be accessible; hence, the identification of BCC through routinely used biochemical tests and treatment with appropriate antibiotics based on antibiotic susceptibility testing armed with knowledge of risk factors associated with BCC infection will enable early recovery and prevent outbreaks.
